# *Dirofilaria* infections in dogs in different areas of Greece

**DOI:** 10.1186/s13071-016-1797-6

**Published:** 2016-09-20

**Authors:** Anastasia Diakou, Emmanouil Kapantaidakis, Androniki Tamvakis, Vassilios Giannakis, Nina Strus

**Affiliations:** 1 Laboratory of Parasitology and Parasitic Diseases, School of Veterinary Medicine, Faculty of Health Sciences, Aristotle University of Thessaloniki, 54124 Thessaloniki, Greece; 2 Department of Marine Sciences, School of the Environment, University of the Aegean, University Hill, 81100 Mytilene, Greece; 3 Animal Health, Elanco Hellas S.A.C.I., Thessaloniki, Greece; 4 Companion Animal CEE, Elanco Animal Health, Ljubljana, Slovenia

**Keywords:** *Dirofilaria immitis*, *Dirofilaria repens*, *Acanthocheilonema reconditum*, Dog, Greece, Prevalence, Risk factors

## Abstract

**Background:**

The nematodes *Dirofilaria immitis* and *D. repens* are enzootic in Greece. In the light of evidence of dirofilariosis spreading to new areas around the world, the aim of the present study was to update and enrich the current knowledge on the prevalence of *Dirofilaria* infections in dogs in Greece, to assess the risk factors of heartworm infection, and to initiate the drawing of the epizootiological map of canine dirofilariosis, investigating *Dirofilaria* infections in five locations along the north-south axis of the country, i.e. municipalities of Thessaloniki, Larissa, Achaia, Attica and Heraklion, associated with the five largest urban centres of Greece.

**Methods:**

Blood samples collected from 750 dogs in total, were examined by the modified Knott’s method and by serology. A questionnaire including information about each examined dog was filled in and statistical analysis of the results was performed using the Chi-square test and a Binary Multiple Univariate Generalized Linear Model.

**Results:**

In total, 31 (4.1 %) out of 750 examined animals were found positive for *D. immitis* by any of the tests applied (Knott’s method and serological examination). Moreover, microfilariae of *D. repens* and *Acanthocheilonema reconditum* were detected by the Knott’s method in 17 (2.3 %) and 10 (1.3 %) of the animals, respectively. At the municipality level, the prevalence of infection was 14, 7, 5.3, 0.7 and 0 % for *D. immitis*, 1, 2, 8.7, 0.3 and 0 % for *D. repens*, and 0, 3, 2.7, 0.7 and 1 % for *A. reconditum* in Thessaloniki, Larissa, Achaia, Attica and Heraklion, respectively. In addition, in three dogs (one each in Thessaloniki, Achaia and Attica) mixed *D. immitis* - *D. repens* infections were detected by the Knott’s method. The area of the country, dog’s usage and age were determined as risk factors for heartworm infection.

**Conclusions:**

Northern areas of Greece have higher *Dirofilaria* prevalence and the prevalence in a western province (Achaia) is recorded for the first time. The mosquito population dynamics recorded in the past is likely to play an important role in the distribution of *Dirofilaria* infections in Greece, and needs further investigation. Similarly, the role of wild reservoirs of filarial parasites in different areas needs to be clarified. Promotion by veterinarians of preventive treatment and compliance by pet owners is essential in all parts of Greece, regardless of the recorded prevalence of infection.

**Electronic supplementary material:**

The online version of this article (doi:10.1186/s13071-016-1797-6) contains supplementary material, which is available to authorized users.

## Background

*Dirofilaria* infections in dogs are caused by the filarial nematode parasites *Dirofilaria immitis* and *Dirofilaria repens*, both transmitted by the bite of infected mosquitoes. *Dirofilaria immitis*, also known as “heartworm”, is the causative agent of dirofilariosis (heartworm disease), one of the most serious parasitic diseases affecting dogs and some other carnivores [1], while *D. repens* parasitizes the subcutaneous and intramuscular connective tissue [2]. Moreover, these parasites, and in particular *D. repens,* have zoonotic implications: *D. immitis* is the causative agent of pneumonic dirofilariosis and *D. repens* can cause subcutaneous or ocular dirofilariosis in humans [3].

Both *D. immitis* and *D. repens* are enzootic in Greece. There are only a few relevant, and, in most cases, outdated epizootiological surveys, most of them conducted in areas of northern Greece, revealing a prevalence ranging from 5 to 34 % and from 18 to 33 % for *D. immitis* and *D. repens*, respectively [4–8]. Only two surveys have been carried out in other areas of the country, i.e. in Attica and Crete (southern Greece), where both species were much less prevalent, i.e. 0.7 and 0 % for *D. immitis* and 0.4 and 0.7 % for *D. repens* in Attica and Crete, respectively [9, 10].

There is also some anecdotal, often contradictory information derived from veterinarians throughout the country, suggesting that heartworm infection is non-existent in some areas or, on the contrary, that cases were confirmed in areas considered as non-enzootic so far. At the same time, there is recent research activity in Europe, introducing the idea of “spreading dirofilariosis” towards northern areas, or to areas that were not considered enzootic until recently [11–15].

In this context, the aim of the present study was to update and enrich the current knowledge on the prevalence of *Dirofilaria* infections in dogs in Greece, to confirm or reject the anecdotal information about the prevalence of heartworm disease in different areas of the country, to assess the risk factors of heartworm infection, and finally, to establish the basis of the current epizootiological map of canine dirofilariosis in Greece.

## Methods

### Study areas

In order to form a basic and representative picture of the prevalence of filarial infection in different areas of Greece, five locations, distributed along the north-south axis of the country, associated with the five biggest urban centres were selected. These were the municipalities of Thessaloniki, Larissa, Attica, Achaia and Heraklion, where the cities of Thessaloniki (40°38′N, 22°56′E), Larissa (39°38′N, 22°25′E), Patras (38°25′N, 21°73′E), Athens (37°97′N, 23°73′E) and Heraklion (35°19′N, 25°8′E) are situated.

### Sampling population

The number of animals to be examined was determined by the number of veterinarians and estimated number of dogs in the area and according to the existing information about the prevalence of *Dirofilaria* spp. in the area (stratified sampling) [16]. The dogs included in the survey were all owned animals, older than 18 months and were not receiving any kind of preventive treatment for dirofilariosis or macrocyclic lactones for other reason. The dogs were sampled during a routine visit to the veterinary practice or were presented by their owners in order to participate in the survey, after notification by the veterinarian. In total, 750 dogs were examined, i.e. 100 from Thessaloniki, 100 from Larissa, 150 from Achaia, 300 from Athens and 100 from Heraklion.

A questionnaire was designed, including information about the age, gender, breed, hair length, movement to other areas and lifestyle (outdoor living, usage) of the dog, and the environment (type of vegetation and existence of ponds or wetlands).

### Samples and examinations

All animals were examined with the consent of their owner. From each dog, 2 ml of blood was collected from a peripheral vein (jugular, cephalic or saphenous) in EDTA tube. The samples were kept refrigerated (4 °C), and sent within a maximum of 5 days to the Laboratory of Parasitology and Parasitic Diseases (Aristotle University of Thessaloniki).

All the samples were examined by two methods: the modified Knott’s method [17] for the detection and identification of microfilariae (first stage larvae, L1) and the serological commercial kit Pet Check® (IDEXX), for the detection of adult *D. immitis* antigen. The microfilariae retrieved by the Knott’s test were identified under light microscopy at 100× and 400× magnifications on the basis of their morphometric (i.e. length and width) and morphological (i.e. anterior and posterior extremities) features [17–19].

### Statistical analysis

Statistical analysis was performed for *D. immitis* infections as they are the most important from a clinical point of view. The risk factors affecting *D. immitis* occurrence were determined using the Chi-square test of independence between the *D. immitis* test results (positive or negative) and a variety of animal characteristics [20]. The Chi-square testing was securely performed as the sample size was big enough (*n* = 750) and thus the average expected frequency was at least 10 for the significance level of α = 0.01 [21]. Moreover, all the categories significant at the *P* < 0.2 level (risk factors) for the Chi-square test, were selected to be entered in a Binary (*D. immitis* infection) Multiple (many risk factors) Univariate (one output variable) Generalized Linear Model (GLM). This procedure can estimate the risk factors while allows the comparison of the percentages of the infected animals within the values of each characteristic (odds ratio). The statistical analysis was implemented using the R package version 3.2.2 [22].

## Results

By the modified Knott’s method, *D. immitis* microfilariae were found in 19 (2.5 %) out of the 750 dogs. At municipality level, *D. immitis* microfilariae were found in 7 (7 %), 5 (5 %), 7 (4.6 %), 0 and 0 of the dogs from Thessaloniki, Larissa, Achaia, Attica and Heraklion, respectively. *Dirofilaria repens* microfilariae were recovered in 17 (2.3 %) of the dogs, i.e. in 1 (1 %), 2 (2 %), 13 (8.7 %), 1 (0.3 %) and 0 of the dogs from Thessaloniki, Larissa, Achaia, Attica and Heraklion, respectively. Moreover, *Acanthocheilonama reconditum* microfilariae were found in 10 (1.3 %) of the dogs in total and 0, 3 (3 %), 4 (2.7 %), 2 (0.7 %) and 1 (1 %) of the dogs from Thessaloniki, Larissa, Achaia, Attica and Heraklion, respectively. In addition, mixed *D. immitis* and *D. repens* infections were revealed by the Knott’s method overall in 3 cases, i.e. in 1 (1 %), 1 (0.7 %), and 1 (0.3 %) of the dogs from Thessaloniki, Achaia and Attica, respectively. The results of the Knott’s method are presented in details in Table 1.

**Table 1:** Microfilariae detected by the modified Knott’s method in different areas of Greece

Municipality (No. of dogs examined)	No. of dogs with *D.i.* (% ± CI)	No. of dogs with *D.r.* (% ± CI)	No. of dogs with *A.r.* (% ± CI)	No. of dogs with *D.i. + D.r.* (% ± CI)
Thessaloniki (*n* = 100)	7 (7.0 ± 5.0)	1 (1.0 ± 2.0)	0	1 (1.0 ± 2.0)
Larissa (*n* = 100)	5 (5.0 ± 4.3)	2 (2.0 ± 2.7)	3 (3.0 ± 3.3)	0
Achaia (*n* = 150)	7 (4.7 ± 3.5)	13 (8.7 ± 4.5)	4 (2.7 ± 2.6)	1 (0.7 ± 1.3)
Attica (*n* = 300)	0	1 (0.3 ± 0.7)	2 (0.7 ± 0.9)	1 (0.3 ± 0.7)
Heraklion (*n* = 100)	0	0	1 (1.0 ± 2.0)	0
Total (*n* = 750)	19 (2.5 ± 1.1)	17 (2.3 ± 1)	10 (1.3 ± 0.8)	3 (0.4 ± 0.5)

*Abbreviations*: *D.i. D. immitis*, *D.r. D. repens*, *A.r. Acanthocheilonema reconditum*, *D.i. + D.r. D. immitis* and *D. repens* mixed infections; *CI* 95 % confidence interval

**Fig. 1:**
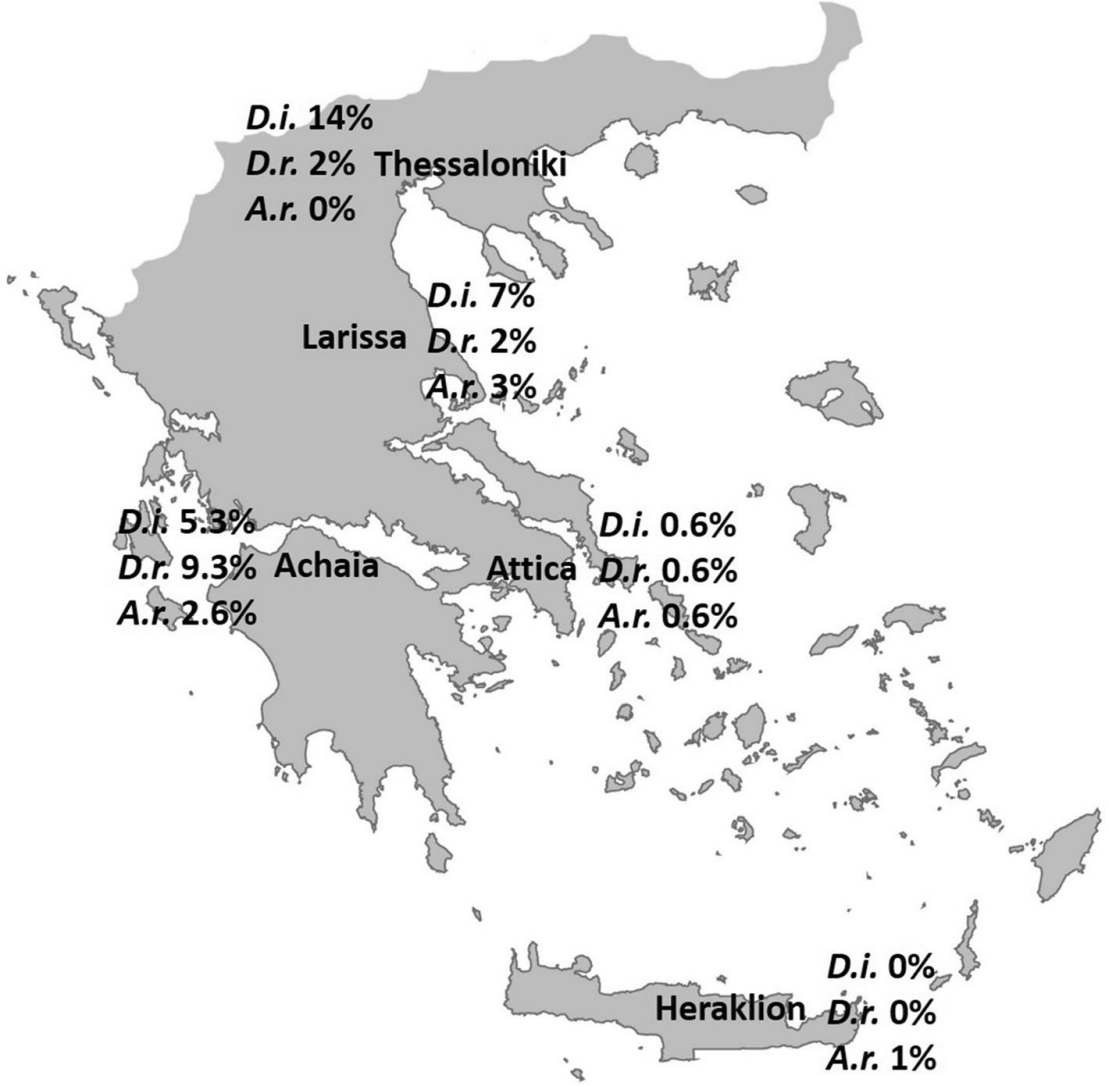
The map of Greece showing the areas of investigation (Municipalities of Thessaloniki, Larissa, Achaia, Attica and Heraklion) and the prevalence of *Dirofilaria immitis* (*D.i.*, as found both in Knott’s and serology), *D. repens* (*D.r.*) and *Acanthocheilonema reconditum* (*A.r.*) infections in each area

*Dirofilaria immitis* antigen was detected by the serological examination in 28 (3.7 %) of the 750 dogs. At municipality level, 14 (14 %), 4 (4 %), 8 (5.3 %), 2 (0.7 %) and 0 dogs were found positive from Thessaloniki, Larissa, Achaia, Attica and Heraklion, respectively. However, three dogs from Larissa, although found negative in serology, were positive for *D. immitis* microfilariae by the Knott’s method, subsequently increasing the number of positive dogs to 7 (7 %) in Larissa and to 31 (4.1 %) overall (Table 2). In 19 out of 31 *D. immitis* positive samples the Knott’s method was in accordance with the serological test. However, in 9 samples, although *D. immitis* antigen was detected in serology, no microfilariae were found. The overall prevalence for each filarial species, in each location of the survey is shown in Fig. 1. None of the positive dogs originated from or had moved to other areas of the country.

**Table 2:** Dogs positive for *Dirofilaria immitis* by at least one method (Knott’s, serology) in different areas of Greece

Municipality	No. of dogs examined	No. of *D. immitis-*positive dogs (% ± CI)
Thessaloniki	100	14 (14.0 ± 6.8)
Larissa	100	7 (7.0 ± 5.0)
Achaia	150	8 (5.3 ± 3.6)
Attica	300	2 (0.7 ± 0.9)
Heraklion	100	0
Total	750	31 (4.1 ± 1.4)

Regarding the statistical analysis of the risk factors of *D. immitis* infection (Table 3), the municipality where the dog lived, its usage and its age were the most important risk factors (Chi-square test of independence for municipality: *χ*^2^ = 40.22, *df* = 4, *P* < 0.001; for usage: *χ*^2^ = 27.51, *df* = 7, *P* < 0.001; for age: *χ*^2^ = 6.09, *df =* 2, *P* = 0.048). On the other hand, factors gender, breed, hair length, body size, living together with other animals, water collections and type of vegetation in the area were not associated with higher risk of infection. The statistically significant characteristics by the Chi-square test were entered in the multiple GLM showing the same statistically significant results (Table 3).

**Table 3:** Assessment of risk factors of *Dirofilaria immitis* infection and results of multiple Generalized Linear Model (GLM) for the risk factors driving *Dirofilaria immitis*

Variable	Odds ratio	95 % CI	GLM (multiple) *P*-value
City			
Thessaloniki *vs* Attica	26.8	6.4–185.5	< 0.001***
Larisa *vs* Attica	11.9	2.6–85.5	0.003**
Achaia *vs* Attica	6.0	1.3–42.1	0.033*
Heraklion *vs* Attica	ns		0.989
Age category			
2–7 *vs* ≤ 2	6.4	1.6–43.0	0.019*
> 7 *vs* ≤ 2	9.5	2.2–67.4	0.007**
Usage			
Guard *vs* Pet	8.2	2.4–33.0	0.001**
Hunter *vs* Pet	5.3	1.6–20.8	0.009**
Dogs			
Yes *vs* No	ns		0.331
Day living			
Outside *vs* Inside	ns		0.356
Night living			
Outside *vs* Inside	ns		0.733
Outside life			
Partially *vs* None	ns		0.464
Exclusively *vs* None	ns		0.363
Water pools			
Yes *vs* No	ns		0.910

*P < 0.05; **P < 0.01; ***P < 0.001

According to this model, a dog living in Thessaloniki, Larissa or Achaia was 26.8, 11.9 and 6 times, respectively, more likely to be infected than a dog living in Attica. Moreover, guard and hunting dogs were almost 8.2 and 5.3 times, respectively, more likely to be infected than pet dogs. Regarding the age, a dog between 2 and 7 years and one older than 7 years were 6.4 and 9.5 times, respectively, more likely to be infected than a dog of an age up to 2 years.

## Discussion

Many European countries are enzootic for *Dirofilaria* infections [23]. During recent years, filarial nematodes seem to have spread in areas previously considered free of these parasites [14]. The expansion of distribution of filarial parasites is attributed to climate change, the increasing abundance of mosquitoes, the expanding movement of the main hosts, i.e. microfilaraemic dogs between different areas and the abundance of wild reservoirs [11]. In this context, it is important to monitor the prevalence and risk factors of filarial infections both in enzootic and in non-enzootic areas in order to ensure a minimum of surveillance of these pathogens that are of both veterinary and medical interest. In Europe, the areas with high prevalence in filarial infections in dogs are located in the south, e.g. Portugal (15.1 %), Spain (2–35 %), southern France (15 %) and Italy (2.3– > 50 % along the Po River Valley) [11, 14, 24–26]. Furthermore, reports of autochthonous cases and prevalence of infection from previously non-enzootic areas are being constantly reported [14].

The findings of the present study are in accordance with previous reports [4–10] and confirm that the prevalence of dirofilariosis is higher in northern than in southern areas of Greece. Our results suggest that the percentage of infected animals lowers progressively towards the south. Indeed, *D. immitis* was found in 14 % of the dogs in the northernmost location (Thessaloniki) and was recorded progressively lower in the locations with smaller latitude, i.e. 7 % in Larissa, 5.3 % in Achaia, 0.6 % in Attica and 0 % in Heraklion. Despite there being a recent confirmed autochthonous case of heartworm infection in a dog in Heraklion (Diakou, unpublished data), it seems that the disease is practically non-existent, at least to date, as both in the present study and in the survey of sheltered stray dogs 20 years ago [10], no *D. immitis*-positive dog was recorded. The prevalence found in Achaia is a new record, in the sense that, to date, filarial infections have never been investigated in this area of Greece (western Greece). However, our communication with the local vets revealed that at least since the 1990s, heartworm infections have been present in the area.

Similarly, *D. repens* infection was also found in higher prevalence in northern and western areas of the country, while in southern areas the occurrence of the infection was very low or even non-existent. Interestingly, there is a recent report of 8 cases of human ocular dirofilariosis due to *D. repens*, in which all patients were residents of northwest Greece [27]. Although this parasite is of known endemicity in areas of southern Europe, it has been shown that infection rates are increasing, both in well-known endemic areas and in northern and eastern areas formerly considered free of the infection [15, 28].

Microfilariae of *A. reconditum* were also found in different areas in the present survey. This is a filarial nematode with global distribution and fleas or lice as intermediate hosts. *Acanthocheilonema reconditum* adults are found beneath the subcutaneous tissues mainly in the limbs and back, and are considered less pathogenic than other filarial nematodes of the dog [29]. The distribution of *A. reconditum* infections as found in the present study reflects the different epizootiological characteristics of this parasite, as its prevalence did not follow the respective for *Dirofilaria* sp. and remained in relatively low levels in all areas (0–3 %). This species has been found in previous surveys in Greece, ranging from 0 to 12 % in different areas [4, 6, 7, 9, 10]. It is worth noting that the highest prevalence (12 %) recorded to date in Greece, was found in sheltered dogs in Heraklion, Crete [10], a fact that could be attributed to the critical role of reservoir dogs in a confined environment, as the closeness to a microfilaraemic animal is crucial for the transmission of infection, which is due to the nature of intermediate hosts [29].

The discordant result of nine samples being antigen positive for *D. immitis* but negative in the Knott’s method was rather expected, as occult heartworm infections are common [30]. The risk of some of these dogs being infected with *Angiostrongylus vasorum* and thus giving a cross reaction to the serological test used here [31] can be considered minimum, as *A. vasorum* is so far considered very rare in Greece [32].

Conversely, in three samples although *D. immitis* microfilariae were detected in the Knott’s method, serology was negative. There are quite a few similar reports of false negative serological tests in the literature [24, 33]. Various factors can lead to a false negative serological test [29, 33], one of them being the antigen-antibody complex formation, that in some cases occurs to such an extent that it does not leave enough antigen circulating to be detected by the serological tests [34]. Consequently, a combination of these two diagnostic tests (Knott and serology) is recommended for higher sensitivity in heartworm diagnosis.

Among the risk factors evaluated here, the area where the dog lived (municipality) was the most determinative factor. The usage of the animal was also very important, thus, guard and hunting dogs were at higher risk of infection, probably as a consequence of the outdoor lifestyle and higher exposure to mosquito bites. Age was also identified as an important risk factor, most likely related to the accumulation of transmission periods and, subsequently, opportunities for an infection to occur in hosts that are not under preventive treatment. Age has been found to be among the recognised risk factors for filarial infection in dogs [35, 36]. However, in some similar studies, no risk factors for filarial infection could be identified [26, 37].

The results of the present study suggest that the situation of heartworm disease in the country has remained quite similar in recent decades, with *D. immitis* and *D. repens* infections being more prevalent in the north compared to the rest of the country, while there is evidence, that in western areas like Achaia, there is an important prevalence of filarial infections. The situation is not the same in Italy, where, until the end of the 1980s, heartworm infection was also mainly a problem in the northern areas of the country, but during recent years its distribution patterns have changed, as more cases of *D. immitis* infection were detected in the south of the country [12].

The distribution of *Dirofilaria* infections in Greece, at first glance, seems unexpected and odd for several reasons: first of all, obviously, the definitive hosts (mainly dogs and other carnivores) as well as the vectors (mosquitoes) are present throughout the country. In addition, the mean temperatures all year round would facilitate faster extrinsic development and a higher number of transmission cycles in the southern parts than in the northern parts of the country. In fact, according a *Dirofilaria* Development Unit (DDU) -based forecast model applied to different areas of Europe, a whole extra month of transmission (i.e. November) is expected in southern Greece, compared to the rest of the areas examined [15]. Attica and the Island of Crete (where the municipality of Heraklion is located) in particular, are among the areas with the second highest yearly average predicted number of *Dirofilaria* generations in Europe, i.e. 8–10 generations, according to the Linear Kriging interpolation [23]. Thus, the recorded distribution of *Dirofilaria* prevalence in northern and southern areas of Greece is contradictory to what is expected based on the models mentioned above, and requires further attempts to explain.

It is known that although temperature is an important factor for the establishment and the prevalence of infection in an area, there are other factors that influence the transmission risk of *Dirofilaria* spp. [38] and, of course, all of these factors are linked to the availability and abundance of vectors (mosquitoes) and reservoirs (definitive hosts). Mosquito population dynamics may be essential in the transmission of pathogens [35, 39]. Mosquitoes are present in all parts of Greece but their populations are not equal, neither in terms of abundance nor stability throughout the country. Indeed, as was revealed by a CO_2_ traps network, sampled twice a month at 106 constant sampling sites throughout Greece, during the summer of 2011, total mosquito populations were significantly higher in northern than in southern Greece [40]. In particular, populations of *Culex* spp. in the region of Central Macedonia were found to be 20 times higher than in the Peloponnese region and even 31 times higher than in Attica region. Also *Aedes* spp. populations in the region of Eastern Macedonia and Thrace, are ten times higher than in the Peloponnese region and western Greece [40].

The existence of wetlands and rice fields, as expected, seems closely related to elevated mosquito populations and, subsequently, higher risk for *Dirofilaria* transmission. Indeed, the bigger and most important wetlands in Greece are located in the north and west of the country [41]. Thus, the map of the wetlands in the country interestingly matches the map of dirofilariosis as illustrated in the present study. Similarly, 91 % of the total rice cultivation areas on a national level are located in northern Greece [42]. Rice field abundance was acknowledged as one of the possible elements contributing to the higher *Dirofilaria* infection rates in certain areas of Portugal [24].

Another factor that should be considered regarding the heartworm geographical distribution in Greece, is the role of wild canids. Although little information is available on the microfilaraemia of these animals, it cannot be excluded that they may contribute to the spreading of the infection [43]. As populations of jackals and wolves are widely distributed in the central and northern part of the country (jackals are also present in Peloponnese), but absent elsewhere [44, 45] the role of these species in the prevalence configuration of heartworm infections in Greece is worth investigating.

## Conclusions

The results of the present study suggest that *Dirofilaria* infections in Greece are more prevalent in the northern areas compared to the southern areas and that the occurrence of *Dirofilaria* infections in the west of the country is not negligible. Moreover, *A. reconditum* infections have been recorded in low prevalence in various areas of the country. Targeted investigations about the role of mosquito populations, and definitive hosts other than the dog, would provide evidence based interpretations for the *Dirofilaria* distribution in Greece. Finally, it is necessary to emphasize that prevention is essential because of both veterinary and medical importance of *Dirofilaria* infections, and should be applied in all dogs and cats in Greece, regardless of the prevalence of infection, considering that conditions permit the completion of the filarial life cycle in all areas of the country.

## Additional file

Additional file 1: Data collected from the questionnaires filled in for each examined dog. (XLSX 91 kb)

## Abbreviations

DDU: *Dirofilaria* Development Unit
GLM: Generalized Linear Model
